# Health-Related Effects of a Short Isometric Exercise Program Integrated into School Physical Education: The Role of Biological Maturation and Baseline Functional Status

**DOI:** 10.3390/healthcare14020161

**Published:** 2026-01-08

**Authors:** Dawid Koźlenia, Rafał Szafraniec, Jakub Jarosz, Leszek Mazur, Jarosław Domaradzki

**Affiliations:** 1 Faculty of Physical Education and Sport, Wroclaw University of Health and Sport Sciences, 51-612 Wrocław, Poland; dawid.kozlenia@awf.wroc.pl (D.K.); jaroslaw.domaradzki@awf.wroc.pl (J.D.); 2 Institute of Sport Sciences, Jerzy Kukuczka Academy of Physical Education in Katowice, 40-065 Katowice, Poland; j.jarosz@awf.katowice.pl; 3 Department of Physical Culture and Health Sciences, Karkonosze University of Applied Sciences, 58-506 Jelenia Góra, Poland; leszek.mazur@kans.pl

**Keywords:** isometric contraction, adolescents, biological age, strength, school settings

## Abstract

**Highlights:**

**What are the main findings?**

- Integrating short isometric exercise bouts into regular physical education did not improve health-related functional or cardiovascular outcomes beyond standard PE alone.
- Improvements in muscular strength and functional capacity were observed over time in both groups, indicating a beneficial effect of regular physical education itself.
- Biological maturation influenced absolute strength levels but did not modify responsiveness to the isometric exercise intervention.

**What is the implication of the main finding?**

- Adding brief isometric exercise to school-based physical education may not provide additional health benefits when regular PE already ensures sufficient physical stimulus.
- Well-designed standard PE programs may be sufficient to support health-related functional development in adolescents.

**Abstract:**

**Objectives:** This study examined whether integrating an isometric exercise program into physical education (PE) lessons influences functional outcomes and cardiovascular risk markers in adolescents beyond the effects of standard PE alone. **Methods:** Boys aged 14–15 years were randomly assigned to an experimental group (EG, *n* = 19) or a control group (CG, *n* = 21). The EG completed a 6-week isometric exercise program integrated into PE lessons, while the CG followed the regular PE curriculum only. The intervention was based on hold isometric muscle actions (HIMA) with progressively increased volume. Anthropometric measures included body height, body mass, and body mass index (BMI). Body composition was assessed using bioelectrical impedance analysis. Functional capacity was evaluated using field-based measures of lower-limb strength and power (isometric mid-thigh pull, standing broad jump, squat jump, and countermovement jump). Systolic and diastolic blood pressure were measured as indicators of cardiovascular health. **Results:** A mixed model ANOVA showed that no significant group × time interactions were observed for body composition, functional outcomes, or blood pressure (all *p* > 0.05). Lean body mass increased over time in both groups (*p* < 0.01). Improvements in isometric mid-thigh pull (*p* < 0.01) and standing broad jump (*p* = 0.01) occurred irrespective of group allocation. Blood pressure remained unchanged. Linear regression revealed that biological maturation did not moderate intervention effects; however, more mature participants demonstrated higher absolute strength levels independent of the intervention (*p* < 0.05). **Conclusions:** The inclusion of an isometric exercise program within PE lessons did not provide additional benefits for health-related functional outcomes beyond standard PE alone. In its current format, isometric exercise does not appear to add sufficient value to justify its implementation as a stand-alone strategy in school-based PE.

## 1. Introduction

Health encompasses not only the absence of disease, but also functional capacity and physical fitness, which are closely linked to metabolic and cardiovascular status [1]. This perspective corresponds to the concept of health-related fitness, which frames physical fitness components—such as muscular strength, cardiorespiratory fitness, and body composition—as clinically and functionally relevant indicators of health, rather than markers of athletic performance [2]. Within this framework, regular physical activity and structured exercise are recognized as key modifiable factors supporting health maintenance and disease prevention across the lifespan. Alarmingly, more than 80% of adolescents worldwide do not meet the WHO guidelines recommending at least 60 min of moderate-to-vigorous physical activity per day [3]. In Poland, the prevalence of overweight among adolescents aged 15–19 has increased from 13% to 20% over the last decade [4], representing a serious risk factor for cardiovascular and metabolic diseases. These trends highlight the importance of school-based physical education as a population-level opportunity to promote regular physical activity and support health-related fitness during adolescence, a critical period for the establishment of long-term health behaviors.

Resistance training has emerged as an effective approach to address these issues by promoting muscular strength, favorable body composition, bone mineral density, and metabolic health [5,6,7]. Importantly, when properly supervised, youth resistance training is both safe and effective, dispelling earlier misconceptions regarding its harmfulness [7,8,9]. Moreover, muscular strength is considered a fundamental capacity underlying other components of fitness, including power, speed, agility, and endurance [6,7]. From a health perspective, muscular strength is increasingly recognized as an important marker of functional capacity and future cardiometabolic health, rather than solely a performance-related attribute [8,9].

Physical education (PE) represents a universal platform for promoting physical activity and health among youth [10]. However, limited lesson duration (typically 45 min) and the necessity to meet curriculum requirements call for strategies that maximize the health-related impact of available time [11]. These constraints necessitate time-efficient strategies that can be realistically integrated into PE lessons while still providing meaningful health-related stimuli. Recently, the concept of microdosing—short, frequent bouts of exercise—has gained attention as a feasible solution [12]. Interventions of this type, including high-intensity interval training (HIIT) and exercise snacks, have demonstrated improvements in aerobic capacity, reductions in fat mass, and decreases in blood pressure among adolescents [13,14]. These findings suggest that brief, structured exercise interventions implemented within PE lessons may contribute meaningfully to adolescent health. These findings suggest that brief, systematically implemented exercise interventions may offer a pragmatic approach to health promotion within school settings.

Within the spectrum of resistance exercise modalities, isometric exercise appears particularly promising. Studies have shown that isometric contractions can induce muscle hypertrophy, enhance static and dynamic strength, and improve performance in selected motor tasks [15,16,17,18,19,20,21]. Isometric contractions are characterized by a lower energy cost compared with concentric actions [22,23,24], while also offering low injury risk and potential analgesic effects, making them applicable in prevention and rehabilitation contexts [25,26]. From a practical perspective, these characteristics align well with the safety, feasibility, and inclusivity requirements of school-based physical education. However, despite growing evidence supporting the effectiveness of isometric exercise in controlled or sport-oriented settings, data on its application within regular PE lessons remain limited [27]. In particular, it is unclear whether short-term isometric training programs of limited duration, such as those realistically implementable within a school curriculum, can meaningfully influence health-related outcomes, including body composition, functional capacity, or blood pressure, in non-athletic adolescent populations. In addition, biological maturation plays a critical role in neuromuscular development and training responsiveness during adolescence [28]. Whether biological maturation and baseline fitness levels modify responsiveness to brief isometric exercise in school settings remains insufficiently explored, yet this information is essential for evaluating the practical value of such interventions as health-oriented educational practices. Research conducted in natural school settings must account for the organizational structure of physical education, in which lessons are delivered to intact classes according to a fixed timetable [9,10,11]. Consequently, school-based interventions are commonly implemented and randomized at the class level, which enhances ecological validity but limits strict individual-level control [10]. At the same time, substantial inter-individual variability in biological maturation and baseline fitness during adolescence justifies examining individual responses within this clustered context [28]. Therefore, school-based studies should balance class-level implementation with the exploration of individual determinants of training responsiveness. Given these gaps, further research is needed to determine whether isometric exercise can serve as a feasible and effective health-promoting strategy within school-based physical education. Therefore, the aim of the present study was to examine whether integrating a brief isometric exercise program into regular PE lessons influences health-related functional outcomes and cardiovascular risk markers in adolescents. Specifically, this study addressed the following research questions: (1) does the inclusion of a short isometric exercise program during regular PE lessons provide additional benefits for functional fitness and blood pressure beyond those achieved through standard PE alone, and (2) are potential training responses influenced by biological maturation status or baseline levels of physical performance? If effective, such an approach could represent a simple, low-cost, and safe method to support adolescent health without disrupting curricular requirements.

## 2. Materials and Methods

This study was designed as a quantitative, school-based controlled trial with repeated measures, aimed at evaluating the effects of a short isometric exercise intervention implemented during regular physical education lessons.

### 2.1. Sample Size

A priori power analysis was conducted using G*Power (G*Power 3.1, Düsseldorf, Germany) [29] to determine the required sample size for detecting a within–between interaction for repeated measures. The analysis specified a medium effect size (f = 0.30), an alpha level of 0.05, a desired statistical power of 0.80, two groups, two repeated measurements. Under these parameters, the minimum required total sample size was 36 participants. In addition, a post hoc power analysis was performed using the final sample size (*n* = 40). Using the same model (within–between interaction), an effect size of f = 0.30, α = 0.05, two groups, two measurements, the achieved statistical power was 0.96. This indicates that the study was sufficiently powered to detect medium-sized interaction effects.

### 2.2. Participants

A total of 56 first-year high-school students were initially screened for eligibility. They were drawn from four intact classes, all following the same physical education curriculum. To avoid contamination between students within the same class, a cluster randomization procedure was applied: two classes were assigned to the experimental condition and two to the control group. Randomization was performed using a simple non-replacement procedure via an online generator (www.randomizer.com).

All participants met the following inclusion criteria: regular attendance in physical education lessons, no medical contraindications to high-intensity exercise (e.g., cardiovascular, respiratory, or musculoskeletal disorders), no participation in structured strength or high-intensity training outside school, and provision of informed consent (with parental consent required for minors). Before the intervention began, several students were excluded due to illness-related absences or participation in extracurricular sport programs during the previous months. During the intervention, additional participants were withdrawn because their attendance during PE lessons fell below the required threshold (absence > 20%). Importantly, all exclusions and withdrawals were unrelated to the intervention protocol itself and were attributable to common school-related factors, including seasonal infections, participation in school exchange programs, travel, or other academic obligations.

In total, 16 students were excluded from the dataset, with an equal number of exclusions in each group (EG: *n* = 8; CG: *n* = 8). The final analytical sample consisted of 40 adolescents (19 in the experimental group and 21 in the control group) who completed all testing sessions. Participation was voluntary, and all students were informed that they could discontinue involvement at any point without consequences. Written consent was obtained from the school administration, parents or legal guardians, and each participant.

### 2.3. Intervention

The intervention was implemented during regular physical education lessons under the supervision of qualified physical education teachers. The isometric exercise program was implemented as a supplementary component within standard physical education lessons and did not replace regular PE activities such as team sports or games. Program adherence was monitored throughout the intervention period by recording attendance during each session. Participants were required to attend at least 80% of scheduled physical education lessons to be included in the final analyses. All planned sessions were delivered as intended, and no meaningful differences in implementation or compliance were observed between classes. The experimental group completed a six-week isometric training program delivered twice per week during regular physical education lessons. A chosen period was selected to reflect a realistic teaching block within the school term and to examine whether meaningful adaptations can be achieved within such a timeframe. All exercises were performed exclusively in an isometric form, requiring students to assume a fixed position and maintain it for a prescribed duration. The program followed a circuit-based structure that remained constant throughout the intervention, while training volume was progressively increased by manipulating the number of circuits, work durations, and rest intervals. In Week 1, students performed two circuits of 10 s holds with 5 s rest periods (total training time: 3 min). This progressed to three circuits in Week 2 (4.5 min). In Weeks 3 and 4, the duration of each exercise increased to 20 s holds with 10 s rest; students completed two circuits in Week 3 (6 min) and three circuits in Week 4 (9 min). Weeks 5 and 6 introduced 30 s holds with 15 s rest, performed in two circuits in Week 5 (9 min) and three circuits in Week 6 (13.5 min). If a student was unable to sustain the full duration—particularly in the final weeks—they were encouraged to complete the hold with one brief pause; if additional pauses were required, the exercise was discontinued to ensure safety and technique integrity. Each session consisted of six isometric exercises performed in the same order: wall sit, isometric push-up hold, hollow body, plank, arch body, and isometric lunges. For the lunges, the prescribed work time was divided equally between the left and right leg without rest between sides (e.g., 10 s represented 5 s per limb). All exercises required no equipment and were selected to target major muscle groups while ensuring feasibility in a school environment. Sessions were supervised by the PE teacher and research personnel, who controlled timing and monitored adherence. Participation was voluntary, and no adverse events were observed. After completing the isometric training circuit, students performed a brief cooldown, consisting of light walking and simple dynamic or breathing-focused movements to reduce acute muscular tension. Immediately after the cooldown, the class resumed the standard physical education lesson according to the regular curriculum. The control group participated exclusively in regular physical education classes following the national curriculum. Their lessons focused on general physical development and included a variety of activities such as team sports (e.g., volleyball, football, basketball), recreational games, athletics elements, rhythmic-movement activities, and other forms of moderate-to-vigorous physical exertion. No additional strength-oriented or isometric training was introduced.

### 2.4. Measurements

#### 2.4.1. Procedures

Morphological and physiological assessments were conducted twice: once during the week preceding the 6-week intervention and again during the week immediately following its completion. All measurements were carried out in indoor sports facilities under standardized conditions. The testing schedule was divided into two separate sessions to ensure consistency and minimize fatigue. On the first testing day, blood pressure was assessed at the beginning of the session after a period of seated rest. Subsequently, anthropometric measurements—including body height, body mass, and body composition—were obtained barefoot and in light clothing. On a second, separate day within the same week, participants completed the physical fitness assessments. These included the jump performance tests and other motor ability measures used in the study. All tests were performed in indoor sports halls between 8:00 a.m. and 1:00 p.m. to ensure uniform environmental conditions. Participants wore standard athletic attire (T-shirts, shorts, sports shoes). The same testing sequence, environmental setup, and researcher instructions were applied during both measurement weeks. This two-day structure was repeated post-intervention, following the same order of procedures.

#### 2.4.2. Blood Pressure Measurements

Blood pressure was assessed using an automated oscillometric device (Omron BP710, Omron Healthcare, Hoffman Estates, IL, USA), following procedures consistent with previous methodological guidelines [30]. An appropriately sized cuff was selected for each participant based on upper arm circumference. Before the measurements, participants rested quietly in a seated position for 10 min. Three readings were taken at 10 min intervals, and the mean value of these three measurements was used for analysis. Blood pressure was measured at baseline, immediately after the intervention period.

#### 2.4.3. Body Morphology

Body height was measured to the nearest 0.1 cm using a professional anthropometer (GPM Anthropological Instruments, DKSH Ltd., Zürich, Switzerland). Measurements were taken with participants standing barefoot, following the recommendations of the International Society for the Advancement of Kinanthropometry (ISAK) [31]. Body mass and body fat percentage (BF%) were assessed using a Tanita Inner Scan V body composition analyzer (model BC-601, Tanita Co., Tokyo, Japan), whose reliability has been previously documented [32]. Prior to testing, students received instructions on the measurement procedures and were asked to empty their bladders, avoid excessive fluid intake, and maintain their typical breakfast habits. All measurements were performed with participants barefoot and shirtless, positioned so that their heels were aligned with the rear electrodes of the scale, lower limbs extended at the knee and hip joints, arms slightly abducted and flexed at the shoulders, elbows straight, and fingers placed on the hand electrodes. Assessments were conducted at least 3 h after the last meal. Body mass index (BMI) was calculated as body mass divided by height squared (kg/m^2^).

#### 2.4.4. Maturity Offset

Biological maturation was assessed using the maturity offset (MO) method, which estimates the number of years an individual is positioned relative to their predicted age at peak height velocity (APHV). The calculation was based on sex-specific equations derived from fundamental anthropometric measurements, following the predictive approach proposed by Moore et al. [33]. The same procedure had been applied previously in related research [34]. For boys, the prediction equation was:

MO=−7.999994+0.0036124×(age×body height)

Maturity offset values were then used to characterize the developmental status of participants within each group. Instead of using fixed biological categories, the distribution of MO scores was divided into quartiles separately for the experimental and control groups. This allowed for a relative classification of maturity level within each sample, reflecting inter-individual variation in growth progression. The resulting quartile-based grouping provided a standardized way to compare maturation differences within and between the study groups without imposing predefined categorical thresholds.

#### 2.4.5. Jump Height

Both the countermovement jump (CMJ) and squat jump (SJ) were performed in accordance with the methodological recommendations of Comfort et al. [35] and Petronijevic et al. [36]. For the CMJ, participants maintained their hands on their hips and executed a rapid downward movement to approximately a 90° knee angle before attempting a maximal vertical takeoff. Emphasis was placed on maintaining consistent technique, ensuring symmetrical push-off and landing patterns. The SJ began from a static 90° knee-flexion position, held for approximately three seconds to eliminate any countermovement, after which participants performed an explosive vertical jump. Each test was performed for three to five attempts, with at least one minute of passive rest between efforts. The order of tasks was randomized to minimize sequencing effects. The highest value obtained for each jump type was retained for subsequent analyses. All jump measurements were collected using a Chronojump contact platform (Chronojump Bosco-System, Barcelona, Spain), whose reliability has been previously validated [37]. The SJ was incorporated into the testing battery to provide additional insight into jump-specific force production.

The eccentric utilization ratio (EUR) was derived by dividing CMJ performance by SJ performance. This metric reflects the athlete’s capacity to effectively use the slow stretch–shortening cycle (SSC). An optimal EUR is typically around 1.1, indicating that CMJ height should exceed SJ height by roughly 10% [38].

#### 2.4.6. Standing Broad Jump

The Standing Broad Jump (SBJ) was assessed by instructing participants to stand behind a marked line and perform a maximal forward jump using a natural arm-swing for momentum, landing simultaneously on both feet [39]. Jump distance was recorded as the straight-line measurement from the take-off line to the nearest heel contact point. Each student performed two attempts, with the longer distance used for analysis. Results were measured with a precision of 0.5 cm.

#### 2.4.7. Isometric Mid-Thigh Pull (IMTP)

Participants performed the IMTP in an upright stance, with the device anchored between the floor and a fixed point positioned at the mid-thigh level according to principles [40]. The height of the attachment point was individually set to reproduce the joint angles typically used during mid-thigh pulling actions. To minimize the influence of grip strength and ensure accurate transfer of force through the lower and upper limbs, lifting straps were applied. Each participant completed three maximal 3 s pulls. Before every attempt, a standardized countdown (“3, 2, 1, pull”) was provided, followed by strong verbal encouragement. Participants were allowed to apply minimal pre-tension only to eliminate slack in the system. The primary outcome variable was peak isometric force (PF), defined as the highest force value recorded during the 3 s maximal contraction. The IMTP was evaluated using the Muscle Meter handheld dynamometer (MAT Assessment, Melbourne, Australia), a portable force-measurement device suitable for field-based strength testing. The system consists of a compact force transducer connected to a digital display unit, which provides real-time feedback and automatically stores peak force values. Adjustable handles and straps were used to standardize body positioning and ensure stability during maximal isometric efforts.

### 2.5. Statistics

All statistical analyses were performed using repeated measures procedures to evaluate within- and between-group differences across time. Descriptive statistics were calculated for all variables and presented as means, standard deviations, and 95% confidence intervals. Prior to hypothesis testing, data distribution was verified using the Shapiro–Wilk test, and assumptions of homogeneity of variances and sphericity were examined using Levene’s and Mauchly’s tests, respectively. No major violations were identified that would preclude the use of parametric procedures. To examine the effects of the intervention, a mixed model ANOVA with one between-subject factor (group: EG vs. CG) and one within-subject factor (time: PRE vs. POST) was applied. For each variable, three effects were tested: (1) main effect of group, indicating whether the groups differed irrespective of time, (2) main effect of time, reflecting overall changes from PRE to POST, and (3) group × time interaction, indicating whether the magnitude or direction of change differed between groups. For each ANOVA model, F-values, *p*-values, and partial eta squared (ηp^2^) were reported as measures of effect size. When significant time effects were observed, Δ-scores (POST–PRE) were calculated to describe the magnitude of change. To further explore whether biological maturity or baseline performance influenced the observed adaptations, linear regression models were conducted. Separate models were estimated for each dependent variable that demonstrated significant change over time (ΔIMTP, ΔSBJ, ΔLBM). Two sets of predictors were examined: (1) maturity offset (MO) predicting the magnitude of changes, (2) baseline values (IMTP, SBJ, LBM) predicting the corresponding Δ-scores. For all regression models, standardized (β) and unstandardized (B) coefficients, standard errors, t-statistics, and *p*-values were reported. The significance threshold for all analyses was set at α = 0.05. Although randomization was performed at the class level, statistical analyses were conducted at the individual level. This approach reflects the natural organization of physical education lessons, which are delivered to intact classes, making individual-level allocation or differentiated programming within the same class impractical. All participants within each class completed the same standardized intervention, and the study was designed to reflect real-world school conditions. Given the small number of classes, the study was not powered to estimate cluster-level effects; therefore, participants were treated as independent observations to preserve statistical power. This approach is consistent with exploratory, school-based intervention studies conducted under natural educational settings. All analyses were performed using standard statistical software (Jamovi (version 2.6.25)).

## 3. Results

In Table 1, descriptive statistics are presented descriptive statistics considering body morphology and physical performance parameters.

For body morphology variables, the mixed model ANOVA for BMI, BW, BF, and LBM showed no significant group × time interactions (all *p* > 0.05). However, LBM demonstrated a significant main effect of time (F = 10.33, *p* < 0.01, ηp^2^ = 0.21), indicating increases over the intervention period irrespective of group. Other anthropometric outcomes remained unchanged.

SBP and DBP showed no significant effects of group or time. The interaction for DBP approached significance (*p* = 0.07), suggesting a possible trend toward differential changes between groups, but this did not meet the threshold for statistical significance. Further, there were no significant main or interaction effects for squat jump SJ or CMJ, indicating that jump height did not differ between groups and did not change across time. For the SBJ, a significant main effect of time was observed (F = 19.58, *p* < 0.01, ηp^2^ = 0.33), suggesting meaningful improvement across measurements in both groups, while neither the group effect nor the interaction reached significance.

EUR values did not show significant main effects or interaction effects (all *p* > 0.50), indicating stable stretch-shortening cycle efficiency throughout the intervention. A significant time effect emerged for IMTP (F = 16.55, *p* < 0.01, ηp^2^ = 0.30), reflecting improvements in lower-limb force for both groups, whereas group and interaction effects were not significant. All results were presented in Table 2.

In the next step of the analysis the linear regression was performed to assess if maturity offset or baseline results predict the magnitude of changes (Δ) in variables changed over time (Table 3).

The regression models examining predictors of MO did not reveal any statistically significant effects. Neither the change in IMTP (ΔIMTP; β = 0.14, *p* = 0.38), the change in standing broad jump distance (ΔSBJ; β = −0.14, *p* = 0.38), nor the change in lean body mass (ΔLBM; β = 0.13, *p* = 0.42) significantly predicted MO values. All standardized coefficients were small, and the associated t-values indicated no meaningful contribution of these variables to the model.

Similarly, the baseline model showed no significant predictors of MO. Neither maximal isometric force at baseline (ΔIMTP; *β* = −0.22, *p* = 0.17), baseline standing broad jump distance (ΔSBJ; *β* = 0.16, *p* = 0.32), nor baseline muscle mass (ΔLBM; *β* = 0.00, *p* = 0.59) was associated with MO outcomes. The very small unstandardized and standardized coefficients further confirm the absence of predictive relationships.

## 4. Discussion

The aim of the present study was to determine the impact of isometric training implemented within physical education (PE) lessons on body morphology, motor performance, and blood pressure in adolescents. The main finding was that the addition of isometric exercise did not result in significant improvements in these outcomes beyond those achieved through regular PE alone. Furthermore, regression analyses indicated that neither biological maturation (maturity offset) nor baseline performance levels predicted the magnitude of changes observed during the intervention period. These findings suggest that, in the applied school-based format, isometric training did not provide an additional stimulus sufficient to enhance health- and fitness-related outcomes beyond those induced by standard PE participation. Taken together, these results indicate that under real-world school conditions, standard PE lessons may already provide a sufficient stimulus to support selected health-related outcomes in adolescents, and that not all additional exercise components necessarily translate into incremental benefits when implemented within the constraints of physical education.

Although isometric exercise has been shown to improve muscular strength and related health outcomes in controlled or athletic settings [17,18,19], its effectiveness appears to be highly dependent on training intensity, volume, and exercise specificity. However, the effectiveness of isometric training appears to depend strongly on training volume, contraction duration, intensity, and contextual factors. In the present study, the intervention was designed to be feasible within the constraints of school PE lessons, which limited both the achievable intensity and total training volume. As highlighted by Lum and Barbosa [18] and Oranchuk et al. [19], long-term adaptations to isometric training typically require prolonged contraction durations or intensities approaching maximal effort. Such conditions may be difficult to implement consistently in a general school setting without compromising safety or lesson structure. Such conditions are difficult to implement consistently in general school settings without compromising safety, inclusivity, or lesson organization. This observation aligns with broader evidence from school-based physical activity research, which suggests that the effectiveness of physically active lessons depends not only on exercise modality but also on feasibility, integration into the curriculum, and overall exposure to moderate-to-vigorous physical activity, rather than on isolated training stimuli alone [41,42].

Evidence from athletic youth populations further supports the importance of training intensity. Dyas et al. [27] demonstrated that substantial improvements in isometric strength and explosive performance occur primarily in response to high-intensity isometric contractions, often exceeding the levels that can be realistically applied during PE lessons. In the present study, the isometric exercises were general in nature, involved submaximal effort, and did not target specific joint angles associated with the outcome measures. Consequently, the applied stimulus may have been insufficient to elicit adaptations beyond those already induced by regular PE activities. This finding underscores a key practical issue: exercise protocols that are effective in athletic or controlled training environments may not yield comparable benefits when translated into real-world educational settings, where safety, inclusivity, and curricular balance are primary considerations [10,43]. In addition, limited exercise specificity may have further constrained the transfer of training effects to dynamic performance measures, as effective transfer of isometric adaptations depends on biomechanical alignment between the training position and the tested task [18,44].

Another factor that may have contributed to the absence of significant between-group differences is the interaction between training-induced adaptations and natural developmental processes. The phenomenon of synergistic adaptation, whereby training effects overlap with maturation-related changes, has been described in youth populations [45,46]. In the present cohort, both groups demonstrated similar improvements over time, suggesting that growth and maturation may have masked subtle intervention-specific effects. This interpretation is supported by the regression analyses, which showed no association between maturity offset or baseline performance and the magnitude of observed changes. Methodologically, this indicates that detecting small intervention effects in adolescent populations may require either higher training doses or longer intervention periods to exceed the background effects of natural development. From a healthcare perspective, this suggests that regular participation in PE during adolescence may support health-related development across a wide range of biological maturity levels, reinforcing the inclusive role of PE as a population-level health intervention.

It is also important to consider the activity profile of the control group. Students in the control condition regularly participated in moderate- to high-intensity activities such as team sports and games during PE lessons. As shown in the meta-analysis by Behringer et al. [47], such activities provide an effective stimulus for improving strength, coordination, and power in youth with low to moderate baseline fitness levels. In addition, dose–response recommendations for youth resistance training indicate that higher intensities and appropriately structured loading are required to elicit further improvements in physical fitness [48,49]. Taken together, these observations suggest that regular PE lessons in the present study already provided a meaningful stimulus, leaving limited scope for additional benefits from a brief, low-volume isometric intervention. Given the class-level randomization applied in this study, statistical analyses were conducted at the individual level, which may have influenced the precision of the estimated effects. This reflects typical school settings, not accounting for potential clustering at the class level, could have resulted in an underestimation of standard errors and a reduction in effective statistical power, particularly for detecting small intervention-related differences [9,10,11]. Therefore, the absence of significant group × time interactions should be interpreted with caution, especially in the context of outcomes showing trends close to statistical significance. In this context, no statistically significant group × time interactions were observed for blood pressure or squat jump performance; near-significant trends were identified for diastolic blood pressure (*p* = 0.07) and squat jump height (*p* = 0.08). While these findings should be interpreted cautiously, they may reflect small effects that were not fully captured due to limited statistical power, short intervention duration, inter-individual variability in responsiveness, and the class-level randomized design. Future studies with larger samples and longer follow-up periods may help clarify whether such trends translate into clinically or functionally meaningful adaptations.

Several limitations should be acknowledged when interpreting the findings of this study. First, the sample consisted exclusively of boys, which limits the generalizability of the results to female adolescents or mixed-sex populations. Second, the relatively narrow age range restricted the ability to examine potential age- or maturation-related differences in responsiveness to isometric training. Third, the modest sample size may have reduced the statistical power to detect smaller, yet potentially meaningful, effects. Additionally, although randomization was conducted at the class level, statistical analyses were performed at the individual level. While this approach reflects the natural organization of physical education lessons and is commonly applied in school-based research, the lack of adjustment for potential clustering effects should be considered a limitation. Further, the study assessed a limited set of physiological and performance outcomes, while other factors—such as neuromuscular adaptations, motivation, or habitual physical activity levels—were not measured. The lack of exercise specificity relative to the outcome measures, as well as the relatively short duration and low volume of the intervention imposed by realistic PE constraints, may have further limited observable adaptations. Moreover, the six-week duration of the intervention, although reflecting realistic constraints of school-based physical education, may have been insufficient to elicit more pronounced adaptations. Future studies should consider longer intervention periods, larger and more diverse samples, and broader outcome measures to better evaluate the role of isometric exercise in school-based physical education. These limitations highlight the need for cautious interpretation and emphasize that the present findings are specific to school-based physical education settings rather than controlled or sport-focused training environments.

## 5. Conclusions

The present study examined the effects of isometric training implemented within physical education (PE) lessons on adolescents’ body morphology, motor performance, and blood pressure. The results indicate that, in the applied school-based format, the addition of isometric exercise did not lead to improvements beyond those achieved through standard PE alone. Furthermore, training responses were not influenced by biological maturation or baseline performance levels, suggesting that the intervention did not provide an additional stimulus under typical PE conditions. These findings should be interpreted in the context of the study limitations discussed above, including the characteristics of the intervention and the study sample. In addition, although no statistically significant group × time interactions were observed, near-significant trends for selected variables (e.g., diastolic blood pressure and squat jump performance) suggest that small effects cannot be entirely excluded and warrant further investigation.

From a practical perspective, these findings suggest that well-structured, regular participation in standard physical education lessons already provides meaningful health- and fitness-related benefits for adolescents. Under the conditions applied in the present study, the addition of short isometric exercise bouts did not result in further measurable improvements. This does not indicate that isometric exercise is ineffective per se, but rather that its implementation within school-based PE may require modification in terms of intensity, duration, or integration with dynamic activities to offer added value. Consequently, PE teachers may prioritize consistent engagement in standard PE activities, while considering isometric exercises as an optional supplementary tool when adapted to specific educational objectives and practical constraints. The present results are specific to real-world school settings and may not directly translate to more controlled or sport-focused training environments. The present results are specific to real-world school settings and may not directly translate to more controlled environments or sport-focused training programs.

## Figures and Tables

**Table 1:** Descriptive statistics of analyzed parameters.

Variable	Experimental			Control		
	Mean ± SD (CI 95%)			Mean ± SD (CI 95%)		
	PRE	POST	Δ	PRE	POST	Δ
BH [m]	1.77 ± 0.06 (1.74–1.8)	1.78 ± 0.06 (1.75–1.81)	0.27 ± 0.26 (0.14–0.40)	1.77 ± 0.09 (1.73–1.81)	1.77 ± 0.09 (1.73–1.81)	0.26 ± 0.25 (0.14–0.37)
BW [kg]	20.10 ± 2.89 (18.70–21.60)	20.20 ± 2.81 (18.80–21.60)	0.04 ± 0.23 (−0.08–0.15)	21.00 ± 3.15 (19.60–22.40)	20.90 ± 3.09 (19.60–22.30)	−0.04 ± 0.25 (−0.15–0.07)
BMI [kg/m^2^]	20.10 ± 2.89 (18.63–21.40)	20.09 ± 2.82 (18.60–21.42)	0.03 ± 0.19 (−0.07–0.12)	20.78 ± 3.16 (19.38–22.20)	20.80 ± 3.07 (19.42–22.10)	−0.05 ± 0.22 (−0.15–0.05)
BF [%]	14.61 ± 4.33 (12.46–16.76)	15.22 ± 4.04 (13.21–17.23)	0.61 ± 1.61 (−0.19–1.4)	16.68 ± 2.93 (15.38–17.98)	16.77 ± 3.18 (15.36–18.18)	0.09 ± 2.51 (−1.02–1.20)
LBM [kg]	50.61 ± 7.14 (47.06–54.16)	52 ± 7.78 (48.13–55.87)	1.39 ± 2.03 (0.37–2.40)	51.68 ± 8.19 (48.05–55.31)	52.14 ± 8.14 (48.53–55.75)	0.455 ± 1.60 (−0.25–1.16)
SBP [mmHg]	123.78 ± 10.34 (118.63–128.92)	122.56 ± 10.99 (117.09–128.02)	−1.22 ± 9.02 (−5.71–3.26)	124.32 ± 9.57 (120.07–128.56)	123.77 ± 10.38 (119.17–128.38)	−0.545 ± 7.79 (−4.00–2.91)
DBP [mmHg]	72.06 ± 7.37 (68.39–75.72)	75.5 ± 8.02 (71.51–79.49)	3.44 ± 9.70 (−1.38–8.27)	76.86 ± 9.61 (72.6–81.12)	74.68 ± 7.73 (71.25–78.11)	−2.18 ± 9.38 (−6.34–1.98)
SJ [cm]	33.33 ± 4.39 (31.14–35.51)	33.89 ± 4.27 (31.76–36.01)	0.56 ± 1.23 (−0.05–1.17)	32.94 ± 4.97 (30.73–35.14)	32.68 ± 4.81 (30.55–34.82)	−0.25 ± 1.54 (−0.93–0.43)
CMJ [cm]	33.3 ± 5.03 (30.8–35.8)	33.74 ± 5.16 (31.17–36.31)	0.44 ± 2.35 (−0.73–1.61)	32.09 ± 5.1 (29.83–34.36)	31.69 ± 5.52 (29.24–34.13)	−0.41 ± 1.96 (−1.27–0.46)
EUR [CMJ /SJ]	1.00 ± 0.108 (0.949–1.06)	0.997 ± 0.101 (0.947–1.05)	−0.01 ± 0.06 (−0.04–0.03)	0.981 ± 0.129 (0.924–1.04)	0.973 ± 0.121 (0.920–1.03)	−0.01 ± 0.09 (−0.05–0.03)
SBJ [cm]	204.00 ± 24.40 (192.00–216.00)	209.00 ± 25.70 (196.00–221.00)	4.56 ± 4.08 (2.53–6.58)	203.00 ± 15.20 (196.00–209.00)	206.00 ± 16.90 (198.00–213.00)	2.77 ± 5.98 (0.12–5.42)
IMTP [N]	127.75 ± 25.73 (114.96–140.55)	132.82 ± 24.12 (120.82–144.81)	5.07 ± 6.63 (1.77–8.36)	119.19 ± 24.82 (108.19–130.2)	123.8 ± 25.01 (112.71–134.89)	4.6 ± 8.1 (1.01–8.19)
MO	1.42 ± 0.58 (1.13–1.71)			1.36 ± 0.64 (1.08–1.65)		

Abbreviations: BF—body fat percentage; BH—body height; BMI—body mass index; BW—body weight; CMJ—countermovement jump; DBP—diastolic blood pressure; EUR—eccentric utilization ratio (CMJ/SJ); IMTP—isometric mid-thigh pull; LBM—lean body mass; MO—maturity offset; SBJ—standing broad jump; SBP—systolic blood pressure; SJ—squat jump.

**Table 2:** Mixed model ANOVA.

Variable	Effect	F	*p*	*p*-eta
BW [kg]	Group	0.28	0.65	0.01
	Time	0.03	0.18	0.04
	Interaction	0.04	0.28	0.04
BMI [kg/m^2^]	Group	0.68	0.41	0.01
	Time	1.16	0.68	0.0
	Interaction	1.42	0.23	0.13
BF [%]	Group	2.74	0.11	0.07
	Time	1.05	0.31	0.03
	Interaction	0.58	0.45	0.01
LBM [kg]	Group	0.06	0.81	0.00
	Time	10.33	<0.01	0.21
	Interaction	2.65	0.11	0.07
SBP [mm/Hg]	Group	0.09	0.77	0.00
	Time	0.44	0.51	0.01
	Interaction	0.06	0.80	0.00
DBP [mm/Hg]	Group	0.86	0.36	0.02
	Time	0.17	0.68	0.00
	Interaction	3.45	0.07	0.08
SJ [cm]	Group	0.30	0.59	0.01
	Time	0.47	0.50	0.01
	Interaction	3.30	0.08	0.08
CMJ [cm]	Group	1.01	0.32	0.03
	Time	0.00	0.96	0.00
	Interaction	1.54	0.22	0.04
EUR [CMJ/SJ]	Group	0.43	0.52	0.01
	Time	0.27	0.60	0.01
	Interaction	0.01	0.94	0.01
SBJ [cm]	Group	0.12	0.73	0.01
	Time	19.58	<0.01	0.33
	Interaction	1.16	0.28	0.12
IMTP [N]	Group	1.26	0.27	0.03
	Time	16.55	0.00 *	0.30
	Interaction	0.04	0.85	0.00

Abbreviations: BF—body fat percentage; BMI—body mass index; BW—body weight; CMJ—countermovement jump; DBP—diastolic blood pressure; EUR—eccentric utilization ratio (CMJ/SJ); IMTP—peak isometric mid-thigh pull; LBM—lean body mass; SBJ—standing broad jump; SBP—systolic blood pressure; SJ—squat jump. * statistically significant *p* < 0.05.

**Table 3:** Linear regression models.

Independent Variable	Dependent Variable	β	SEβ	B	SEB	*p*
MO	ΔIMTP	0.14	0.16	0.01	0.01	0.38
	ΔSBJ	−0.14	0.16	−0.02	0.02	0.38
	ΔLBM	0.13	0.16	0.04	0.05	0.42
Baseline	ΔIMTP	−0.22	0.16	−0.75	0.54	0.17
	ΔSBJ	0.16	0.16	0.61	0.60	0.32
	ΔLBM	0.24	0.16	1.01	0.67	0.14

Abbreviations: IMTP—peak isometric mid-thigh pull; LBM—lean body mass; MO—maturity offset; SBJ—standing broad jump.

## Data Availability

Data for the current study will be available upon reasonable request from the corresponding author.

## Abbreviations

The following abbreviations are used in this manuscript:

ANOVA	analysis of variance
APHV	age at peak height velocity
BF	body fat percentage
BH	body height
BMI	body mass index
BW	body weight
CC BY	Creative Commons Attribution license
CG	control group
CI	confidence interval
CMJ	countermovement jump
DBP	diastolic blood pressure
EG	experimental group
EUR	eccentric utilization ratio (CMJ/SJ)
G*Power	statistical power analysis software
HIIT	high-intensity interval training
IMTP	isometric mid-thigh pull
ISAK	International Society for the Advancement of Kinanthropometry
LBM	lean body mass
MAT	Muscle Meter (device name)
MO	maturity offset
MVPA	moderate-to-vigorous physical activity
N/kg	newtons per kilogram
PE	physical education
PF	peak force
PRE	pre-intervention
POST	post-intervention
RPE	rating of perceived exertion
SBJ	standing broad jump
SBP	systolic blood pressure
SD	standard deviation
SSC	stretch–shortening cycle
WHO	World Health Organization
Δ	change score (POST–PRE)
ηp^2^	partial eta squared
